# Diminished Mortality from Consumption

**Published:** 1852-03

**Authors:** 


### DIMINISHED MORTALITY FROM CONSUMPTION.

In the "Quarterly Summary of the Transactions of the College of Physicians of Philadelphia" just published, we find the following remarks on the efficacy of cod-liver oil in consumption:

Dr. Wood called the attention of the College to the diminished per centage of deaths from consumption in the city of Philadelphia and the surrounding districts, as shown by the tables of mortality recently presented to the College by Dr. Ruschenberger, and published in the last number of its Summary of Transactions. Dr. Wood was inclined to attribute this diminution in the deaths from consumption mainly to the general use of cod-liver oil by individuals threatened with that disease, or in whom its premonitory symptoms had already exhibited themselves. We know of no other general influence to which the diminished mortality could be referred. He remarked, however, that it would be too soon to congratulate ourselves upon having discovered a means by which the fatality of consumption can be effectually and permanently controlled. It is an acknowledged fact, that the cod-liver oil has the power, in some instances, of arresting the progress of tuberculization without entirely eradicating the predisposition to it; consequently, the diminution in the deaths from consumption of the past year may be made up in an increased mortality from this source, exhibited in the mortuary tables of some later period.

Dr. Coates thought that prolongation of life and strength, particularly an average prolongation, ought to be considered as necessarily involving a real increase cf cures. No cure could be effected, in any disease, without the powers of nature. He believed that it was now a settled point in medicine, that tubercles of the lungs, though such a terrible destroyer of the human species, always made an attempt, or had a tendency, to cure; that death was produced by accumulation of disease, and by exhaustion of the powers of life; and that some recoveries really take place. If this be correct, all depends on the prolongation of existence, and the retention of the powers of the constitution. An average prolongation of life, and of a portion of the vital powers, could not, therefore, by the commonest rules, fail to add to the average and real number of *bona fide* recoveries.

